# Simultaneous determination of diosmin and hesperidin in combined pharmaceutical preparation by synchronous fluorescence spectrofluorimetric method

**DOI:** 10.1098/rsos.240268

**Published:** 2024-07-24

**Authors:** H. Askar, M. E. Fathy, M. M. Tolba, Fatma A. Aly, Mohammed E.-S. Metwally

**Affiliations:** Pharmaceutical Analytical Chemistry Department, Faculty of Pharmacy, Mansoura University, Mansoura 35516, Egypt

**Keywords:** diosmin, hesperidin, synchronous spectrofluorimetric method, combined tablets, greenness

## Abstract

A sensitive, rapid and green synchronous spectrofluorimetric method was developed to simultaneously analyze a binary mixture of diosmin (DSM) and hesperidin (HSP). The RSFI of both medications was measured in methanol at Δ*λ* of 100 nm. The results indicated that specific experimental factors had an impact on these intensities. The optimization and thorough examination of these parameters were conducted. The plots of synchronous fluorescence intensity-concentration for DSM and HSP were found to be linear within the concentration ranges of 0.5–5.0 µg ml^−1^ and 0.2–3.0 µg ml^−1^, respectively. The detection limits for DSM and HSP were 0.107 µg ml^−1^ and 0.048 µg ml^−1^, respectively. The limits of quantification were 0.323 µg ml^−1^ and 0.144 µg ml^−1^ for DSM and HSP, respectively. The method outlined in this study was successfully used to determine the quantities of both drugs present in commercially available mixed tablets. The results obtained using this method were subsequently compared to those of a comparison method. Greenness assessment of the suggested procedure was accomplished by applying the GAPI method. Consequently, the recommended method can be used in the routine quality control analysis of the two cited drugs with minimum harmful effect on the environment as well as the individuals.

## 1. Introduction

Phytochemicals, which are plant-derived chemicals, have recently played a significant role in many fields, especially nutraceuticals, cosmetics, functional foods and pharmaceuticals. Flavonoids, a class of phenolics, are the predominant phytochemicals derived from plants that exhibit preventive properties against the development of osteoporosis, cancer and cardiovascular problems. In addition, these substances have demonstrated antiviral, anti-inflammatory, antioxidant, antiallergic and antibacterial properties [1].

**Figure 1.**
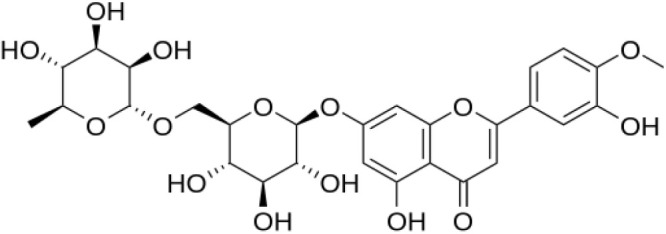
Structure of diosmin (DSM).

**Figure 2.**
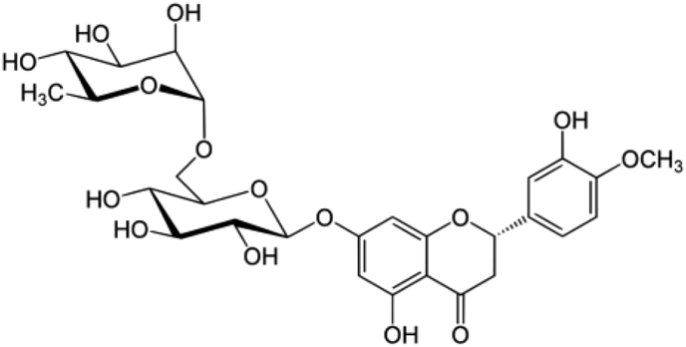
Structure of hesperidin (HSP).

The compound DSM(7-[[6-O-(6-deoxy-α-L-mannopyranosyl)-β-D-glucopyranosyl]oxy]-5-hydroxy-2-(3-hydroxy-4-methoxyphenyl)-4H-1-benzopyran-4-one) (figure 1) is a flavone [2], whereas HSP (5-hydroxy-2-(3-hydroxy-4-methoxyphenyl)-4-oxo-4H-chromen-7-yl rutinoside) (figure 2) is a flavanone [2]. The British Pharmacopoeia (BP) has approved both DSM and HSP [3]. Various methods have been documented for the simultaneous determination of DSM and HSP in biological fluids, dosage forms and plant extracts. The methods used in these reports were spectrophotometric [4,5], chromatographic [6–15] and chemometric [16]. Fluorometric analysis methods demonstrated high sensitivity and selectivity in detecting various substances. However, there is only one spectrofluorimetric method mentioned in the literature that can simultaneously estimate DSM and HSP [17]. In the presence of Tris buffer of pH 8.0 and 8.5 for DSM and HSP, respectively, a ternary complex was formed with Tb^3+^ in the comparison method [17]. The fluorescence quenching was measured at 549 and 494 nm using 275 and 248 nm as excitation wavelengths for DSM and HSP, respectively. The quantitative measurement of Tb^3+^ fluorescence quenching was used to simultaneously estimate DSM and HSP in plasma and combined dosage forms due to the formation of the complex. Our method was found to be straightforward, cost-effective and timesaving, with no need for elaborate derivatization steps or complicated instruments.

Due to their high native fluorescence, we are motivated to develop a spectrofluorimetric technique to measure both drugs simultaneously. There was a significant similarity between the normal emission fluorescence spectra of DSM and HSP. To address this issue, synchronous fluorescence spectroscopy (SFS) was used to quantify the relative synchronous fluorescence intensities (RSFI) at 280 and 390 nm for DSM and HSP, respectively. The SFS technique was developed to simultaneously determine both medications in their combined pharmaceutical tablets.

The advantages of SFS over normal fluorescence spectroscopy are high selectivity, simple spectra and low interference [18]. In a single run, SFS is considered a simple method for quantitative determination and effectively obtaining results due to its narrow and sharp spectrum [19].

## 2. Experimental

### 2.1. Materials and solvents

All used solvents and reagents were of analytical grade.

— A pure sample of diosmin (DSM) was kindly provided by Alamriya company (Alexandria, Egypt).
— A pure sample of Hesperidin (HSP) was kindly provided by Sedico Pharmaceutical Co. (6^th^ October City, Egypt).

Purities of diosmin and hesperidin were certified to be 99.44 and 98.54%, respectively. They were used as provided.

— Daflon® 500 mg tablets: diosmin 450 mg, 50 mg of hesperidin (Batch # 31601) (Servier Egypt Industries Limited, 6^th^ October City, Giza, Egypt), were purchased from the local market (commercial source).
— Disodium hydrogen phosphate and citric acid were provided from ADWIC, El Nasr Pharmaceutical Chemicals, Egypt. NaOH and HCl were also obtained from the same company.
— Methanol was obtained from Sigma-Aldrich (Louis, USA).
— Mcllvaine's buffer, 0.15 M (pH 4.0), was prepared when proper quantities of 0.15 M aqueous solutions of both citric acid and disodium hydrogen phosphate were mixed. The necessary pH was adjusted using a pH meter, and NaOH and HCl were used to obtain buffer solutions with a pH range of 2.0–10. At room temperature, the buffer solutions were stable.

### 2.2. Apparatus

Measurements and fluorescence spectra were recorded using a Cary Eclipse spectrofluorometer (Agilent Technologies, USA) with the Xenon flash lamp provided. The slit width was 5 nm, and the high voltage mode was used (800 v). A smoothing factor of 15 was used. The SF spectra were determined at wavelengths of 280 nm and 390 nm for DSM and HSP, respectively. Fluorescence data managing software, FL WINLAB, Version 4.00.02 (Copyright 2001, Perkin Elmer, UK) was used on a computer connected to the spectrofluorometer. A Sonix IV model-SS101H 230 (USA) was utilized for sonication. In addition, the pH of the buffer solutions was examined using a Consort NV P901 digital pH Meter (Belgium), and standard buffers were used for calibration.

### 2.3. Standard solutions

A 100 µg ml^−1^ solution of DSM was prepared by diluting 400 µg ml^−1^ of DSM. The solution was made by dissolving the required amount in 5 ml of 0.1 N NaOH using a 25 ml volumetric flask and then adding methanol until reaching the mark. Sonication was used to accelerate the dissolution process. A solution of HSP with a concentration of 100 µg ml^−1^ was prepared using the same method as DSM. Both drugs' stock solutions were kept in a refrigerator and remained stable for ten days.

## 3. Procedure

### 3.1. Calibration curves

A series of 10 ml volumetric flasks were filled with aliquots of the DSM and HSP standard solutions covering the working concentration range shown in table 1. Subsequently, methanol was added to achieve the desired dilution level. By scanning both monochromators with 10 nm excitation and emission windows and a constant wavelength difference (Δ*λ*) of 100 nm, SF spectra of the solutions were captured. The SF spectra's intensities for DSM and HSP were determined to be 280 and 390 nm, respectively. The experiment was conducted simultaneously with a blank one. The calibration curves were obtained by plotting the RSFI against the final drug concentrations (µg ml^−1^). An alternative was to derive the corresponding regression equations.

**Table 1.** Analytical performance data for the determination of DSM and HSP in pure forms using the proposed method.

parameter	DSM	HSP
concentration range (µg ml^−1^)	0.5–5.0	0.2–3.0
correlation coefficient	0.9997	0.9998
slope	145.420	236.317
intercept	28.871	31.630
limit of detection (LOD) (µg ml^−1^)	0.107	0.048
limit of quantification (LOQ) (µg ml^−1^)	0.323	0.144
*S_y/x_*	5.906	4.790
*S_a_*	4.695	3.407
*S_b_*	1.538	1.888
%RSD	1.141	1.262
%Error	0.431	0.476

*S_y/x_* = Standard deviation of the residuals.

*S_a_* = Standard deviation of the intercept.

% RSD = Relative standard deviation.

*S_b_* = Standard deviation of the slope.

% Error = %RSD/√n.

*n* = 7.

### 3.2. The synthetic mixture procedure

A series of 10 ml volumetric flasks were filled with aliquots of DSM and HSP standard solutions in a 9 : 1 (w/w) ratio. The solutions were then mixed thoroughly with methanol to dilute them to the desired volume. The recommended action was then carried out under the ‘Calibration Curve.’ The relevant concentrations were calculated using the calibration curves or corresponding regression equations after measuring the RSFI.

### 3.3. Combined commercial tablet procedure

Ten Daflon tablets have been weighed, ground and thoroughly blended. A quantity of the grained tablets was measured and placed into a small conical flask equal to 9.0 mg DSM and 1.0 mg HSP (in ratio of 9 : 1). Afterwards, 5 ml of 0.1 N NaOH and 50 ml of methanol were used to be ultrasonically extracted for 30 min. After being filtered, the extract was transferred into a 100 ml volumetric flask. A small amount of methanol was used to wash the conical flask. The same volumetric flask was used to collect the washings, which were then completed using methanol to the mark. Aliquots of the working concentration ranges were placed into 10 ml volumetric flasks. The suggested procedure listed under ‘Calibration Curves’ was performed. The tablets' nominal content was determined utilizing either the associated regression equations or the previously calculated calibration curves.

## 4. Results and discussion

Our proposed SFS method was found to be an accurate and precise green analytical method. In addition, it was a time- and cost-effective procedure. In terms of green analysis, the method is regarded as a good alternative to the reported methods using large amounts of organic solvents and non-degradable hazardous chemicals.

### 4.1. Synchronous fluorescence spectra of DSM and HSP

**Figure 3.**
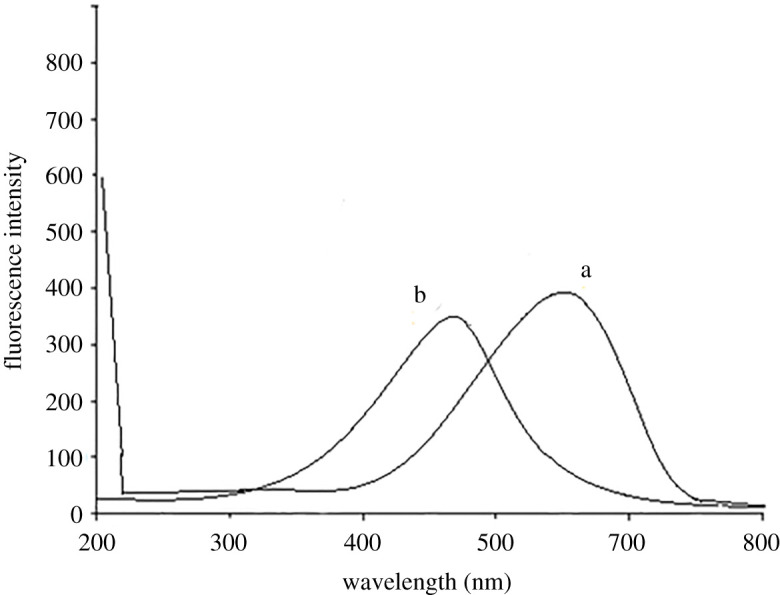
Emission fluorescence spectra of (*a*) DSM (1.5 µg ml^−1^) at 549 nm after excitation at 275 nm. (*b*) HSP (1.0 µg ml^−1^) at 480 nm after excitation at 245 nm.

The DSM exhibited its maximum fluorescence intensity at 549 nm after excitation at 275 nm, while HSP demonstrated its maximum fluorescence intensity at 480 nm after excitation at 245 nm. An overlap was observed in the emission fluorescence spectra of DSM and HSP (figure 3). Therefore, it has been challenging to determine DSM and HSP simultaneously via direct measurement, and identifying these substances in their co-formulated preparations was more difficult.

**Figure 4.**
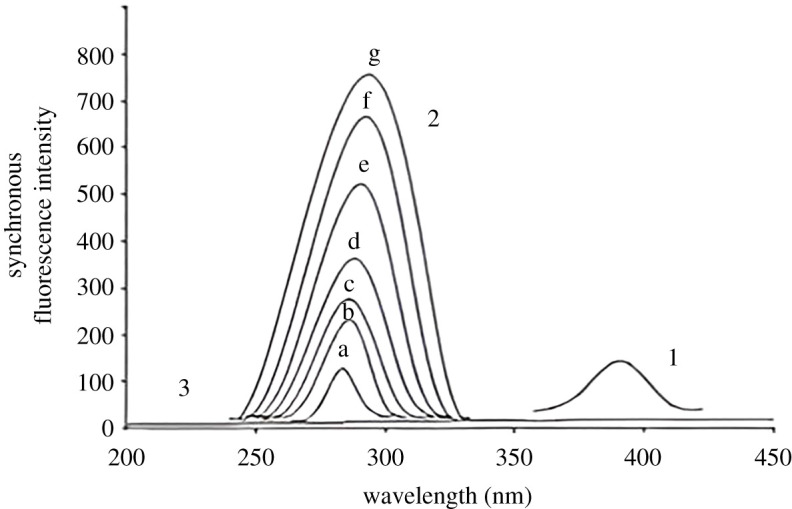
Synchronous fluorescence spectra of (1) HSP (0.5 µg ml^−1^) (2) *a–g*, DSM (0.5, 1.5, 1.8, 2.5, 3.5, 4.5 and 5.0 µg ml^−1^) (3) blank.

**Figure 5.**
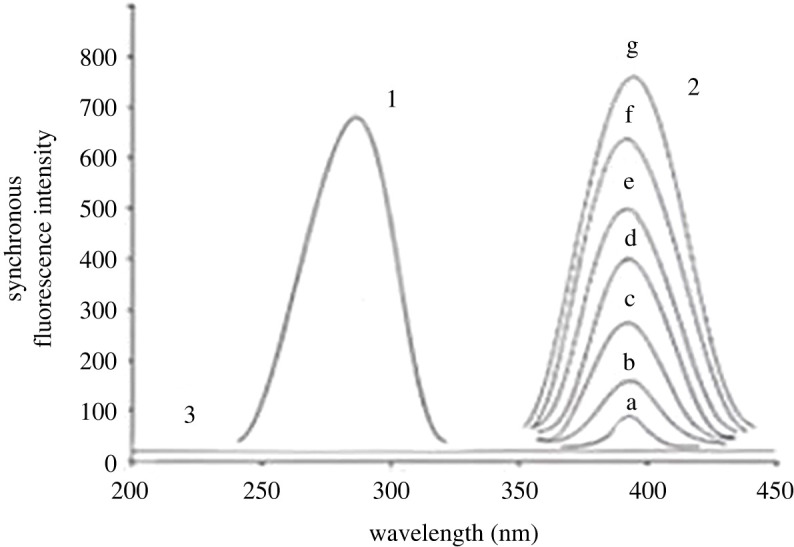
Synchronous fluorescence spectra of (1) DSM (4.5 µg ml^−1^) (2) *a–g*, HSP (0.2, 0.5, 1, 1.5, 2.0, 2.5 and 3.0 µg/ ml^−1^) (3) blank.

Recording the synchronous fluorescence spectra of both DSM and HSP was essential for addressing the issue. After removing the blank value, no overlap was observed between them. The spectra of the blank synchronous and constant concentration of HSP (0.5 µg ml^−1^) were observed along with the SF spectra at 280 nm for various DSM concentrations (figure 4). Figure 5 displays the SF spectra of different HSP concentrations at 390 nm in the presence of spectra of the blank synchronous and constant concentration of DSM (4.5 µg ml^−1^).

### 4.2. Optimization of experimental conditions

The performance of the proposed method was influenced by various experimental parameters, which were subsequently optimized and thoroughly investigated. While holding all other variables constant, each of these factors was individually changed. The diluting solvent type, pH and Δ*λ* were examples of these factors.

### 4.3. Selection of optimum Δ*λ*

**Figure 6.**
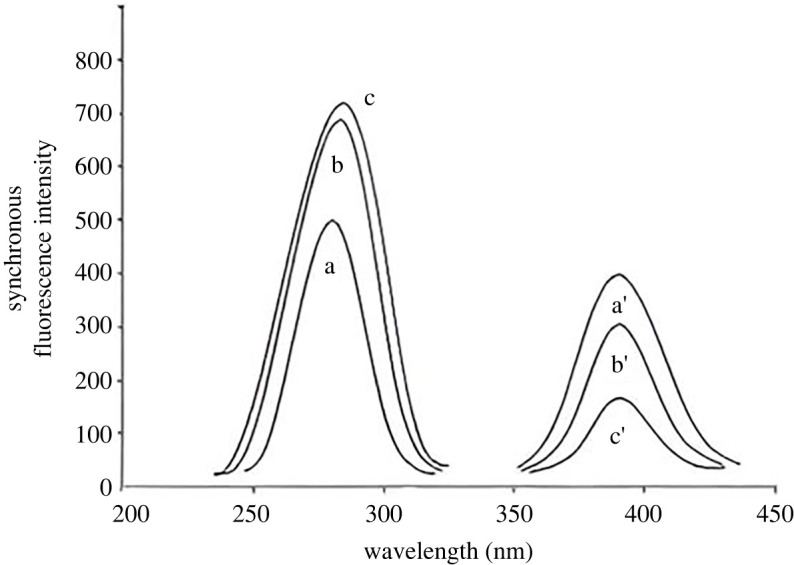
Synchronous fluorescence spectra of DSM and HSP at different Δ*λ*. Where: (*a*, *b* and *c*) for DSM at Δ*λ* of 90, 100 and 110 nm, respectively. (*a′*, *b′* and *c′*) for HSP at Δ*λ* of 90, 100 and 110 nm, respectively.

The optimal value of Δ*λ* value is a critical factor in determining the synchronous fluorescence scanning technique's features, sensitivity and resolution. It has a direct impact on signal strength, bandwidth and spectral shape. This led to the examination of a broad range of Δ*λ* (20, 40, 60, 80, 100 and 120 nm). For DSM, a difference in wavelength (Δ*λ*) below 100 nm resulted in a low sensitivity of the synchronous fluorescence intensity. However, a difference in wavelength of Δ*λ* ≥ 100 nm yielded nearly the same sensitivity. For HSP, Δ*λ* below 100 nm provided very high synchronous fluorescence intensity. However, it simultaneously prevented the estimation of both studied medications (9 DSM: 1 HSP), while Δ*λ* of more than 100 nm led to low sensitivity. The optimal wavelength for separating the mixture of HSP and DSM was determined to be 100 nm due to its ability to accurately detect the desired sensitivity and eliminate any interference caused by the spectrum generated by each drug in the mixture (figure 6).

### 4.4. Selection of optimum pH

Using McIlvaine's buffer, which covers the pH range of 2.0 to 10.0, the impact of pH on the RSFI of the two compounds was investigated. It has been found that the use of a buffer has no impact or may even decrease RSFI. Therefore, no buffer was utilized at any point during the trial to maintain methodological simplicity.

### 4.5. Effect of diluting solvent

**Figure 7.**
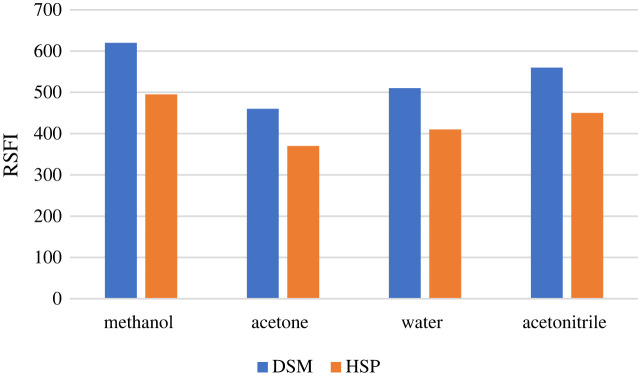
Effect of diluting solvent on relative synchronous fluorescence intensity for DSM (4 µg ml^−1^) and HSP (2 µg ml^−1^).

The solvent dilution was carried out using acetone, methanol, water and acetonitrile. Methanol demonstrated the highest RSFI among the other solvents, specifically for DSM and HSP. Therefore, during the investigation, methanol was the diluting solvent (figure 7).

### 4.6. Validation of the method

The procedure's validity was assessed per ICH Q2 guidelines regarding precision, accuracy, selectivity, linearity and range [20].

### 4.7. Linearity and range

The regression analysis demonstrated a linear relationship between RSFI values and the concentrations of medications within the specified ranges outlined in table 1. The standard deviation of the intercepts (*S_a_*), the residuals (*S_y/x_*) and the slopes (*S_b_*) were used to statistically evaluate the regression lines, so the validity of the approach was demonstrated (table 1). The small values of the figures indicate the high precision and low point scattering observed around the calibration curves.

### 4.8. Limits of quantification (LOQ) and limits of detection (LOD)

In accordance with ICH Q2 recommendations [20], the lowest values at which the calibration curves were nonlinear were used to calculate the limits of quantification (LOQ). To determine the limits of detection (LOD), we utilized the minimum concentrations of analytes that reliably demonstrate identification. The LOD and LOQ of DSM and HSP, as obtained by the SFS method, are summarized in table 1. The following equations were used to calculate LOQ and LOD in accordance with ICH Q2R1 [20] recommendations:

$$\mathrm{LOQ}=\frac{10S_{a}}{b}$$

$$\mathrm{and}\;\mathrm{LOD}=\frac{3.3S_{a}}{b},$$

where *S_a_* is the standard deviation of the calibration curve regression line intercept, and *b* is the slope.

### 4.9. Accuracy

To evaluate the precision of the proposed method (table 2), authentic samples of DSM and HSP were determined across the concentration ranges listed in table 1. The results obtained as well as the comparison method's results showed a good agreement [17]. As depicted in table 2, the student's *t*-test and variance ratio *F*-test results [21] indicated no discernible differences between the two approaches' accuracy and precision performance.

**Table 2.** The synchronous spectrofluorimetric method is applied to determine the studied drugs in pure forms. Figures between parenthesis are the tabulated *t* and *F* values at *p* = 0.05 [21].

parameters	concentration taken (µg ml^−1^)	concentration found (µg ml^−1^)	% found^a^	
			proposed method	comparison method [17]
DSM	0.5	0.503	100.58	100.66
	1.5	1.473	98.17	101.84
	1.8	1.796	99.76	101.33
	2.5	2.504	100.16	
	3.5	3.515	100.42	
	4.0	4.065	101.63	
	5.0	4.945	98.90	
*X*′			99.95	101.28
±SD			±1.14	± 0.59
*T*		1.87 (2.31)		
*F*		3.71 (19.33)		
**HSP**	0.2	0.196	98.10	98.24
	0.5	0.501	100.18	99.28
	1.0	1.009	100.90	98.73
	1.5	1.517	101.10	
	2.0	1.961	98.04	
	2.5	2.511	100.44	
	3.0	3.006	100.20	
*X′*			99.85	98.75
±SD			±1.26	±0.52
*T*		1.42 (2.31)		
*F*		5.86 (19.33)		

^a^Each result is the average of three separate determinations.

**Figure 8.**
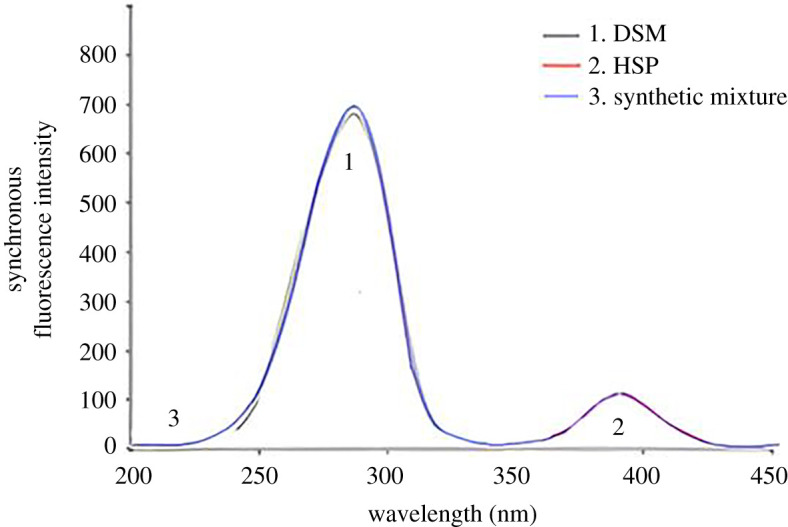
Synchronous fluorescence spectra of (1) DSM (4.5 µg ml^−1^) (2) HSP (0.5 µg ml^−1^) (3) synthetic mixture of DSM (4.5 µg ml^−1^) and HSP (0.5 µg ml^−1^).

The developed approach was used to simultaneously determine DSM and HSP in synthetic combinations with varying doses of both medications in a 9 : 1 (w/w) ratio (figure 8). The relative synchronous fluorescence intensities were assessed for both medications. DSM's RSFI was measured at 280 nm, with HSP showing no contribution. HSP's RSFI was tested at 390 nm, with DSM showing no contribution. The linear regression equations of the calibration curves were used to calculate the concentrations of both medications in the synthesized mixture. Table 3 shows the results, which indicate the level of accuracy achieved by the procedure.

**Table 3.** Application of the proposed method for determination of the studied drugs in their synthetic mixtures.

sample	concentration taken (µg ml^−1^)		concentration found (µg ml^−1^)		% found^a^		comparison method [17]	
	DSM	HSP	DSM	HSP	DSM	HSP	DSM	HSP
DSM and HSP mixture	1.8	0.2	1.803	0.199	100.18	99.39	99.11	100.64
	2.7	0.3	2.712	0.297	100.45	99.04	99.05	100.05
	4.5	0.5	4.502	0.496	100.04	99.24	99.65	99.88
*X*`					100.22	99.22	99.27	100.19
± s.d.					± 0.21	± 0.18	±0.33	±0.40
*T*					2.13(2.78)	2.13(2.78)		
*F*					2.51(19)	5.16(19)		

^a^Each result is the average of three separate determinations.

### 4.10. Precision

The proposed methods were used to assess the determination of three concentrations, with three replicates of each concentration, in intra-day and inter-day assays conducted over three consecutive days. The relative standard deviations were determined to be minimal, indicating a high level of repeatability and intermediate precision of the proposed method (table 4).

**Table 4.** Precision of the proposed method for determination of DSM and HSP raw materials.

concentration taken (μg ml^−1^)	% found^a^	% RSD	% error
**DSM**			
** Intra-day**			
1.8	100.43 ± 0.45	0.44	0.26
2.7	100.88 ± 0.41	0.40	0.23
4.5	99.22 ± 0.19	0.19	0.11
** Inter-day**			
1.8	99.75 ± 0.28	0.29	0.17
2.7	100.35 ± 0.33	0.33	0.19
4.5	100.70 ± 0.27	0.27	0.15
**HSP**			
** Intra-day**			
0.2	99.32 ± 0.79	0.80	0.46
0.3	101.08 ± 0.69	0.68	0.39
0.5	99.42 ± 0.38	0.38	0.42
** Inter-day**			
0.2	100.22 ± 0.53	0.53	0.30
0.3	100.68 ± 0.29	0.29	0.17
0.5	100.67 ± 0.61	0.61	0.35

^a^Each result is the average of three separate determinations.

### 4.11. Robustness

No many factors affected the robustness of the proposed method. To estimate the method robustness, triplicate assays were conducted for the previously mentioned synthetic mixture (9 DSM: 1 HSP). The difference in wavelength (Δ*λ*) was susceptible to ±2 nm. Upon these slight alterations, accepted robustness of this new proposed method was proved due to non significance of the difference between the measured signals.

### 4.12. Selectivity

To evaluate the selectivity of the proposed method for the analysis of co-formulated tablets containing DSM and HSP, the interference effect of various pharmaceutical excipients such as microcrystalline cellulose, magnesium stearate, gelatin, talc and glycerol was studied. Synthetic mixture containing DSM (4.5 µg ml^−1^) and HSP (0.5 µg ml^−1^) in ratio (9 DSM: 1 HSP) was analysed as a representative example with the proposed method in the presence of one of the pharmaceutical additives (20 µg ml^−1^) [22]. It was found that no significant interference effect was observed by the studied excipients on the results of the method (table 5).

**Table 5.** Effect of presence of commonly used excipients on the determination of synthetic mixture of DSM (4.5 µg ml^−1^) and HSP (0.5 µg ml^−1^) in ratio (9 DSM: 1 HSP) as a representative example.

additive (20 µg ml^−1^)	% recovery	
	DSM	HSP
microcrystalline cellulose	98.76 ± 0.15	99.48 ± 0.45
magnesium stearate	100.82 ± 0.18	99.27 ± 0.49
gelatin	98.87 ± 0.18	100.05 ± 0.66
talc	100.71 ± 0.49	101.01 ± 0.12
glycerol	99.310.19	101.32 ± 0.33

### 4.13. Pharmaceutical applications

**Figure 9.**
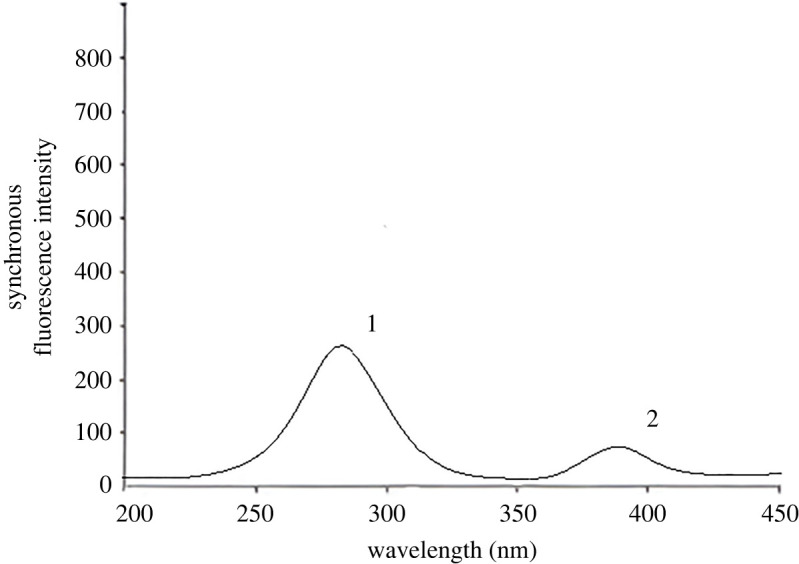
Synchronous fluorescence spectra of (1) DSM (1.8 µg ml^−1^) and (2) HSP (0.2 µg ml^−1^) in their co-formulated tablet.

Daflon®, a tablet containing both medications under study, was analysed using the proposed approach (figure 9). Table 6 shows the successful recovery values from testing Daflon® tablets for DSM and HSP. In addition, *t*-test and *F*-test [21] were applied to statistically compare the obtained outcomes with the comparison method [17] as illustrated in table 6. There was a perfect harmonization between the results of both proposed and comparison methods, which indicated high levels of accuracy and precision.

**Table 6.** Application of the proposed method for determination of the studied drugs in their co-formulated tablet.

preparation	concentration taken (µg ml^−1^)		concentration found (µg ml^−1^)		% found^a^		comparison method [17]	
	DSM	HSP	DSM	HSP	DSM	HSP	DSM	HSP
Daflon® 500 mg Tablets	1.8	0.2	1.802	0.201	100.11	100.18	100.40	99.34
(DSM 450 mg + HSP 50 mg/Tablet)	2.7	0.3	2.702	0.301	100.09	100.19	101.00	99.10
Batch # 31601	4.5	0.5	4.520	0.499	100.44	99.82	100.12	99.71
*X′*		100.21	100.06	100.51	99.38			
± SD		± 0.20	±0.21	±0.45	± 0.31			
*T*					1.04 (2.78)	2.13 (2.78)		
*F*					5.23 (19)	(19)		

**®** Product of Servier Egypt Industries Limited.

Figures between parenthesis are the tabulated *t* and *F* values at *p* = 0.05 [21].

^a^Each result is the average of three separate determinations.

### 4.14. Greenness assessment

The importance of greenness of analytical methods is human protection from chemicals having hazardous effects. In the green analytical method, there is no consumption of excessive energy, no production of harmful waste and toxic organic solvents can't be used. Techniques for greenness assessment of analytical methods include Analytical eco-scale score, National Environmental Methods Index (NEMI) and the Green Analytical Procedure Index (GAPI) [23]. The proposed method which was used recently, the GAPI method, was confirmed to be green, eco-friendly and safe for the environment [24].

A pictogram of the GAPI method is applied for assessment of the effect of each stage of an analytical method on the environment using colour scale of three levels: green, yellow and red, indicating low, medium and high impact on the environment [24].

**Figure 10.**
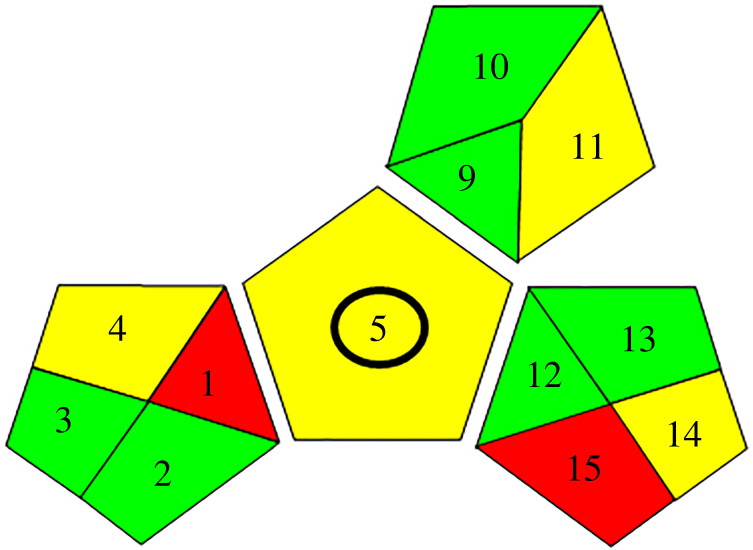
Greenness assessment results of the proposed method.

Figure 10 indicates fulfilling GAPI major criteria except for fields 1,15 (red) because of the off-line sampling and no treatment of the waste, respectively. And fields 4,5,14 (yellow) which related to storage under normal conditions, carrying out the sampling procedure and the formation of 10 ml waste per sample, respectively. Field 11 was also coloured yellow because of using methanol in mobile phase. GAPI was applied for simultaneous estimation of DSM and HSP in their combined tablets; the outcomes were coloured yellow, showing simple preparation (filtration) and the usage of methanol solvent. The results proved that the proposed technique was safe to the environment and humans. Also, it was a green method.

## 5. Conclusion

The two medications were simultaneously estimated using a green, sensitive and simple approach in a binary mixture of both studied drugs. Without addition of any buffer, DSM could be determined in the presence of HSP using a synchronous spectrofluorimetric approach and vice versa in ratio (9 DSM: 1 HSP) at Δ*λ* of 100 nm, so it could be applied in quality control laboratories. In addition, the co-formulated dosage forms of both medications could be analysed using the synchronous spectrofluorimetric approach without excipient interference and the results obtained were favourably compared to those obtained with the comparison method. Precise measurements were obtained for low concentrations, specifically 0.5 µg ml^−1^ for DSM and 0.2 µg ml^−1^ for HSP. In addition, the suggested approach is cost-effective and time-saving, as it eliminates the requirement for complex derivatization procedures or sophisticated instruments. Finally, the method was proved to be green using the Green Analytical Procedure Index (GAPI).

## Data Availability

Data are available at Dryad Digital Respiratory: https://doi.org/10.5061/dryad.70rxwdc5w [25].

## References

[1] Tiwari BK, Brunton NP, Brennan C. 2013 Handbook of plant food phytochemicals: sources, stability and extraction. New York, NY: John Wiley & Sons.

[2] Martindale GJ. 2014 The complete drug reference 38^th^ edition. J. Forensic Leg. Med **28**, 54. (10.1016/j.jflm.2014.10.001)

[3] Commission BP. 2019 British pharmacopoeia 2019. Volume I and V-A. London, UK: Stationery Office.

[4] Bennani I, Chentoufi MA, El Otmani IS, Cheikh A, Bamou N, El Karbane M, Bouatia M. 2020 Development and validation of two spectrophotometric methods for simultaneous determination of diosmine and hesperidin in mixture and their applications. J. Appl. Pharm. Sci. **10**, 100-107. (10.7324/JAPS.2020.104013)

[5] Mowaka S, Mohamed D. 2013 Kinetic spectrophotometric determination of flavonoids using alka-line potassium permanganate.

[6] Piponski M, Stoimenova TB, Topkoska M, Stefov S, Piponska M, Serafimovska GT. 2018 Development and validation of a fast and simple RP-HPLC method for the determination of diosmin and hesperidin in combined tablet dosage form. Maced. J. Chem. Chem. Eng. **37**, 127-134. (10.20450/mjcce.2018.1448)

[7] Szymański M, Młynarek D, Szymański A, Matławska I. 2016 Simultaneous determination of diosmin and hesperidin in pharmaceuticals by RPLC using ionic liquids as mobile phase modifiers. Iran. J. Pharm. Res **15**, 141. 27610154 PMC4986112

[8] Mishra G, Srivastava VK, Tripathi A. 2013 Analytical method development and validation for assay of diosmin and hesperidin in combined tablet dosage form by RP-HPLC. Int. J. Pharm. Life Sci. **4**, 2834-2839.

[9] Shawky E. 2012 Development and validation of an HPTLC method for the simultaneous determination of diosmin and hesperidin in different citrus fruit extracts and pharmaceutical formulations. J Planar Chromatogr TLC. **25**, 138-144. (10.1556/JPC.25.2012.2.9)

[10] Šatínský D, Jägerová K, Havlíková L, Solich P. 2013 A New and Fast HPLC Method for Determination of Rutin, Troxerutin, Diosmin and Hesperidin in Food Supplements Using Fused-Core Column Technology. Food Anal. Methods **6**, 1353-1360. (10.1007/s12161-012-9551-y)

[11] Xu J, Xu C, Liu B. 2013 HPLC Simultaneous Determination of Four Flavone Glycosides in Menthae Haplocalycis Herba during Different Harvest Periods. Chinese J. Pharm. Anal **33**, 2077-2081.

[12] Saeidi I, Hadjmohammadi MR, Peyrovi M, Iranshahi M, Barfi B, Babaei AB, Dust AM. 2011 HPLC Determination of Hesperidin, Diosmin and Eriocitrin in Iranian Lime Juice Using Polyamide as an Adsorbent for Solid Phase Extraction. J. Pharm. Biomed. Anal. **56**, 419-422. (10.1016/j.jpba.2011.05.015) 21683540

[13] Asghari A, Barfi B, Barfi A, Saeidi I, Ghollasi MF, Peyrovi M, Beig Babaei A. 2014 Comparison between Conventional Solid Phase Extraction and Its Simplified Method for HPLC Determination of Five Flavonoids in Orange, Tangerine, and Lime Juice Samples. Acta Chromatogr **26**, 157-175. (10.1556/AChrom.26.2014.1.12)

[14] Xue Y, Qing LS, Yong L, Xu XS, Hu B, Tang MQ, Xie J. 2019 Determination of Flavonoid Glycosides by UPLC-MS to Authenticate Commercial Lemonade. Molecules. **24**, 3016. (10.3390/molecules24163016) 31434256 PMC6719059

[15] Guo P, Yan W, Han Q, Wang C, Zhang Z. 2015 Simultaneous Quantification of 25 Active Constituents in the Total Flavonoids Extract from Herba Desmodii Styracifolii by High-performance Liquid Chromatography with Electrospray Ionization Tandem Mass Spectrometry. J. Sep. Sci. **38**, 1156-1163. (10.1002/jssc.201401360) 25620156

[16] Sayed NW. 2009 Application of Chemometric Methods for Simultaneous Determination of Diosmin and Hesperidin in Pharmaceutical Preparations. Egyptian J Pharm Sci **50**, 1-12.

[17] Mohamed D, Tawakkol SM. 2013 Fluorimetric determination of diosmin and hesperidin in combined dosage forms and in plasma through complex formation with terbium. Bull. Fac. Pharmacy, Cairo Univ **51**, 81-88.

[18] Chen G-Z, Huang X-Z, Xu J-G, Zheng ZZ, Wang Z-B. 1990 The methods of fluorescence analysis. Sci. Beijing **323**, 241-251.

[19] Patra D, Mishra AK. 2002 Recent developments in multi-component synchronous fluorescence scan analysis. TrAC Trends Anal. Chem. **21**, 787-798. (10.1016/S0165-9936(02)01201-3)

[20] ICH Harmonized Tripartite Guideline. 2005 Validation of analytical procedures: text and methodology. Q2 1, 5. See http://www.bioforum.org.il/Uploads/Editor/karen/q2_r1_step4.pdf 11590294

[21] Miller JC, Miller JN. 2005 Statistics and chemometrics for analytical chemistry, 5^th^ edn. Saddle River, NJ: Prentice Hall.

[22] Derayeaa SM, Omara MA, Hammada MA, Hassanb YF. 2017 Application of surface plasmon resonance of citrate capped silver nanoparticles for the selective determination of some fluoroquinolone drugs. J. Appl. Pharm. Sci. **7**, 016-024.

[23] Gamal M, Naguib IA, Panda DS, Abdallah FF. 2021 Comparative study of four greenness assessment tools for selection of greenest analytical method for assay of hyoscine N-butyl bromide. Anal. Methods **13**, 369-380. (10.1039/D0AY02169E) 33404016

[24] Płotka-Wasylka J. 2018 A new tool for the evaluation of the analytical procedure: Green Analytical Procedure ndex. Talanta **181**, 204-209. (10.1016/j.talanta.2018.01.013) 29426502

[25] Askar H et al. 2024 Data from: Simultaneous determination of diosmin and hesperidin in combined pharmaceutical preparation by synchronous fluorescence spectrofluorimetric method. Dryad Digital Repository. (10.5061/dryad.70rxwdc5w) PMC11265867 39050722

